# Online dashboard and data analysis approach for assessing COVID-19 case and death data

**DOI:** 10.12688/f1000research.24164.1

**Published:** 2020-06-08

**Authors:** Hector Florez, Sweta Singh

**Affiliations:** 1 Universidad Distrital Francisco Jose de Caldas, Bogota, Colombia; 2 Savitribai Phule Pune University, Pune, India

**Keywords:** COVID-19, SARS-CoV-2, Data analysis, Mathematical model

## Abstract

The 2019-2020 global pandemic has been caused by a disease called coronavirus disease 2019 (COVID-19). This disease has been caused by the Severe Acute Respiratory Syndrome coronavirus-2 (SARS-CoV-2). By April 30 2020, the World Health Organization reported 3,096,626 cases and 217,896 deaths, which implies an exponential growth for infection and deaths worldwide. Currently, there are various computer-based approaches that present COVID-19 data through different types of charts, which is very useful to recognise its behavior and trends. Nevertheless, such approaches do not allow for observation of any projection regarding confirmed cases and deaths, which would be useful to understand the trends of COVID-19. In this work, we have designed and developed an online dashboard that presents actual information about COVID-19. Furthermore, based on this information, we have designed a mathematical model in order to make projections about the evolution of cases and deaths worldwide and by country.

## Introduction

Coronavirus disease 2019 (COVID-19) is caused by Severe Acute Respiratory Syndrome coronavirus 2 (SARS-CoV-2), which is a virus strain that causes respiratory illness. This virus was identified in December, 2019 and its first infection case was reported on December 30, 2019 in Wuhan city located in Hubei providence, China ^1^. The World Health Organization (WHO) recognized the disease as a pandemic on March 11 2020 ^2^. The WHO have reported the following numbers of cases and deaths worldwide: January 31, 9,847 cases and 213 deaths ^3^; February 29, 85,961 cases and 2,941 deaths ^4^; March 31, 754,933 cases and 36,522 deaths ^5^; and April 30, 3,096,626 cases and 217,896 deaths ^6^. These reports show an increment of cases in February of 872.97%, in March of 878.22%, and in April of 410.18%, as well as an increment of deaths in February of 1,387.26%, in March of 1,241.82%, and in April of 596.61% ^7^. Based on these percentiles, we can observe an exponential growth of the disease.

Based on this previous data, it is important to analyze the evolution of the disease in order to make decisions that tackle the growth rate of cases and deaths. Therefore, in this work, we have designed and developed an online dashboard which presents two types of results: (1) actual information about the COVID-19 using different deployment strategies; and (2) based on the actual information, a projection in order to estimate the future behavior of the disease calculated worldwide and by country. To achieve this projection, we have designed a mathematical model based on previous data reported by the WHO.

## Methods

### Implementation

To analyze the current state of COVID-19, we have created an online information system called *COVID-19 Dashboard*, which is accessible from: https://covid19.itiud.org/. For the design of *COVID-19 Dashboard*, Unified Modeling Language (UML) ^8^ was used allowing the description of the software based on the Object Oriented Paradigm ^9, 10^. It has been developed using: a) PHP 7.4.5 ( https://www.php.net/) supported by Apache 2.4.43 ( https://www.apache.org/); b) Bootstrap 4.4.1 ( https://www.getbootstrap.com/) for responsive front-end; c) jQuery 3.3.1 ( https://www.jquery.com/) as JavaScript library.

*COVID-19 Dashboard* presents full information about COVID-19 focusing on cases and deaths worldwide and by country. In addition, it includes a mathematical model in order to calculate projections for future cases and deaths. The data source used in *COVID-19 Dashboard* is taken from the WHO ( https://covid19.who.int/) and World Population Review ( https://www.worldpopulationreview.com/). The first data set is accessed by *COVID-19 Dashboard* through the URL https://dashboards-dev.sprinklr.com/data/9043/global-covid19-who-gis.json. This information is in JSON format; then, the system includes the algorithms to decode this format before processing the information. The second data set is downloaded in CSV format and included in the persistence layer of the system.This project deploys different graphics to analyze the information from different perspectives ^11^. These graphics are generated based on the JavaScript library Chartkick 2.7.2 ( https://www.chartkick.com/), which is supported by Google Charts ( https://developers.google.com/chart).

To perform the projection for estimating future cases and deaths, we developed a mathematical model focused on a quadratic equation ^12^ based on data of present day and 30 previous days. With the quadratic equation, projected cases and deaths for the next days up to 90 days are calculated. The quadratic equation obtained for each projection is deployed supported by the JavaScript library Mathjax ( https://www.mathjax.org/).

**Figure 1.**
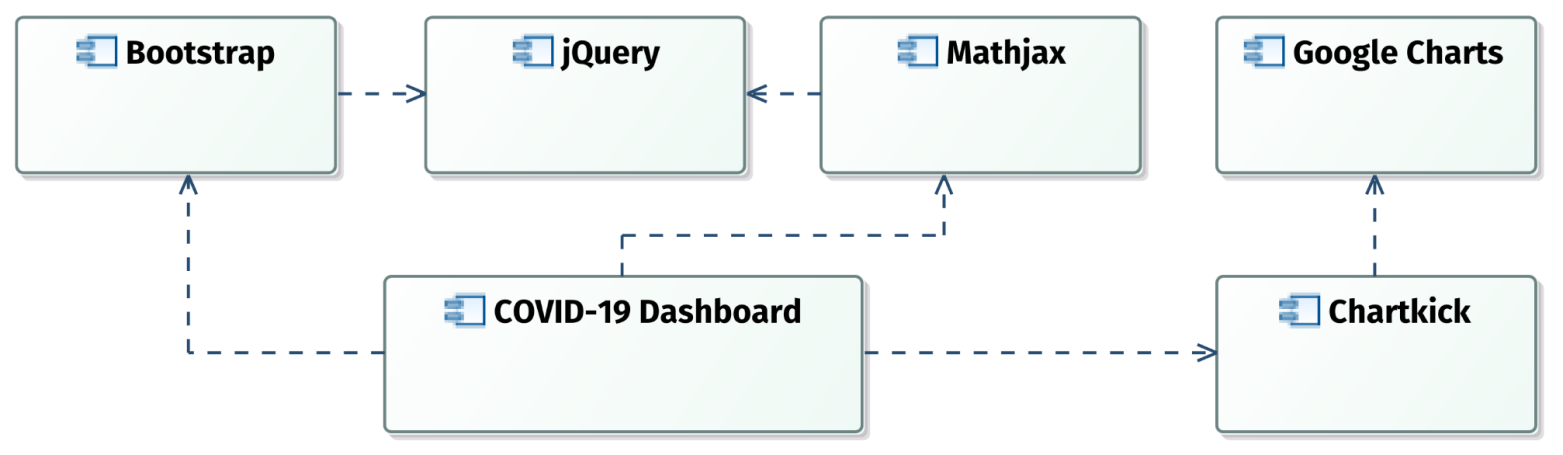
*COVID-19 Dashboard* components.

Figure 1 presents the components of the system, where all mentioned libraries are connected to *COVID-19 Dashboard*. The conceptual models of *COVID-19 Dashboard* have been created using the modeling tool UML Designer 9.0.0 ( http://www.umldesigner.org/). The selected Integrated Development Environment (IDE) was Eclipse-PHP 2020-03 ( https://www.eclipse.org/) and the server used to host the project was Debian Linux Server 10.4 ( https://www.debian.org/) ^13^.

### Modeling projections for cases and deaths

For the mathematical model, let three points *P*_1_ = ( *x*_1_, *y*_1_), *P*_2_ = ( *x*_2_, *y*_2_), and *P*_3_ = ( *x*_3_, *y*_3_) such that *y*_1_ corresponds to number of cases or deaths of the date 30 days prior to the present day, *x*_1_ = 0, *y*_2_ corresponds to number of cases or deaths of the date 15 days prior to the present day, *x*_2_ = 15, *y*_3_ corresponds to number of cases or deaths of the present day, and *x*_1_ = 30. Based on the quadratic equation defined as *f* ( *x*) = *ax* ^2^ + *bx* + *c*, let the equation system presented in ( 1), ( 2), and ( 3).

$$y_{1}=ax_{1}^{2}+bx_{1}+c\;(1)$$

$$y_{2}=ax_{2}^{2}+bx_{2}+c\;(2)$$

$$y_{3}=ax_{3}^{2}+bx_{}+c\;(3)$$

Since *x*_1_ = 0 from ( 1):

$$y_{1}=c\;(4)$$

Subtracting ( 2) from ( 4):

$$y_{1}-y_{2}=-ax_{2}^{2}-bx_{2}\;(5)$$

Isolating the variable *b* from ( 5):

$$b=\frac{y_{1}-y_{2}+\;ax_{2}^{2}}{-x_{2}}\;(6)$$

Subtracting ( 2) from ( 3):

$$y_{3}-y_{2}=a\left(x_{3}^{2}-x_{2}^{2}\right)+b\left(x_{3}-x_{2}\right)\;(7)$$

Replacing *b* in ( 7):

$$\begin{array}{l} y_{3}-y_{2}=a\left(x_{3}^{2}-x_{2}^{2}\right)+\frac{y_{1}-y_{2}+ax_{2}^{2}}{-x_{2}}\left(x_{3}-x_{2}\right)\\ y_{3}-y_{2}=a\left(x_{3}^{2}-x_{2}^{2}\right)+\frac{\left(y_{1}-y_{2}\right)\left(x_{3}-x_{2}\right)}{-x_{2}}-ax_{2}\left(x_{3}-x_{2}\right)\\ y_{3}-y_{2}=a\left(\left(x_{3}^{2}-x_{2}^{2}\right)-x_{2}\left(x_{3}-x_{2}\right)\right)-\frac{\left(y_{1}-y_{2}\right)\left(x_{3}-x_{2}\right)}{x_{2}}\;\left(8\right) \end{array}$$

Isolating the variable *a* from ( 8):

$$a=\frac{y_{3}-y_{2}}{\left(\left(x_{3}^{2}-x_{2}^{2}\right)-x_{2}\left(x_{3}-x_{2}\right)\right)}+\frac{\left(y_{1}-y_{2}\right)\left(x_{3}-x_{2}\right)}{x_{2}\left(\left(x_{3}^{2}-x_{2}^{2}\right)-x_{2}\left(x_{3}-x_{2}\right)\right)}\;\left(9\right)$$

Finally, isolating the variable *c* from ( 4):

$$c=y_{1}\;\left(10\right)$$

Therefore, based on *a*, *b*, and *c* applied to the quadratic equation, the projected cases and deaths are calculated. In addition, calculated quadratic equations will be adjusted when new information is reported by the WHO. Furthermore, *COVID-19 Dashboard* offers a projection for 90 days; nevertheless, for some countries, the quadratic equation could rise to a maximum point; in these cases, the algorithms will stop calculating and thus the projected days will be lower than 90 days.

Consequently, *COVID-19 Dashboard* allows analyzing confirmed cases, deaths, and mortality rates as well as projecting cases and death deploying information in: a) geographic charts to understand the country distribution of the disease, b) bar charts to recognize the most impacted countries, c) column charts to present the evolution of the disease over time, and d) line charts to estimate the behavior of the disease.

### Operation

*COVID-19 Dashboard* is an online responsive system; therefore, it is accessible for users via a web browser through desktops, laptops, smart phones, and tablets. Its front-end can automatically adjust to any screen resolution in order to deploy the information in the best possible way.

## Results

*COVID-19 Dashboard* is able to process the data provided by the WHO related to COVID-19 rates and World Population Review related to the current world population. This system deploys up-to-date information as follows:

- *Cases and deaths by country.* This information is presented in two ways: geographic charts and bar charts in order to recognize the most impacted countries.
- *Cases and deaths by country per million people.* This information is also presented by geographic and bar charts.
- *Mortality rate by country.* For this information, the mortality rate is calculated for each country and is presented by geographic and bar charts.
- *Cases and deaths by date worldwide and by selected country.* This information is calculated based on the daily information reported by each country. This is presented in two column charts. The first column chart presents the daily cases, while the second column chart presents the cumulative cases.
- *Projected cases and deaths worldwide and by selected country.* This information is calculated using the quadratic equation obtained through the developed mathematical model (as above). It is presented using a line chart with two series. The first presents the actual cases or deaths for the last 31 days. The second presents the projected cases or deaths up to 90 days after the last day reported.

**Figure 2.**
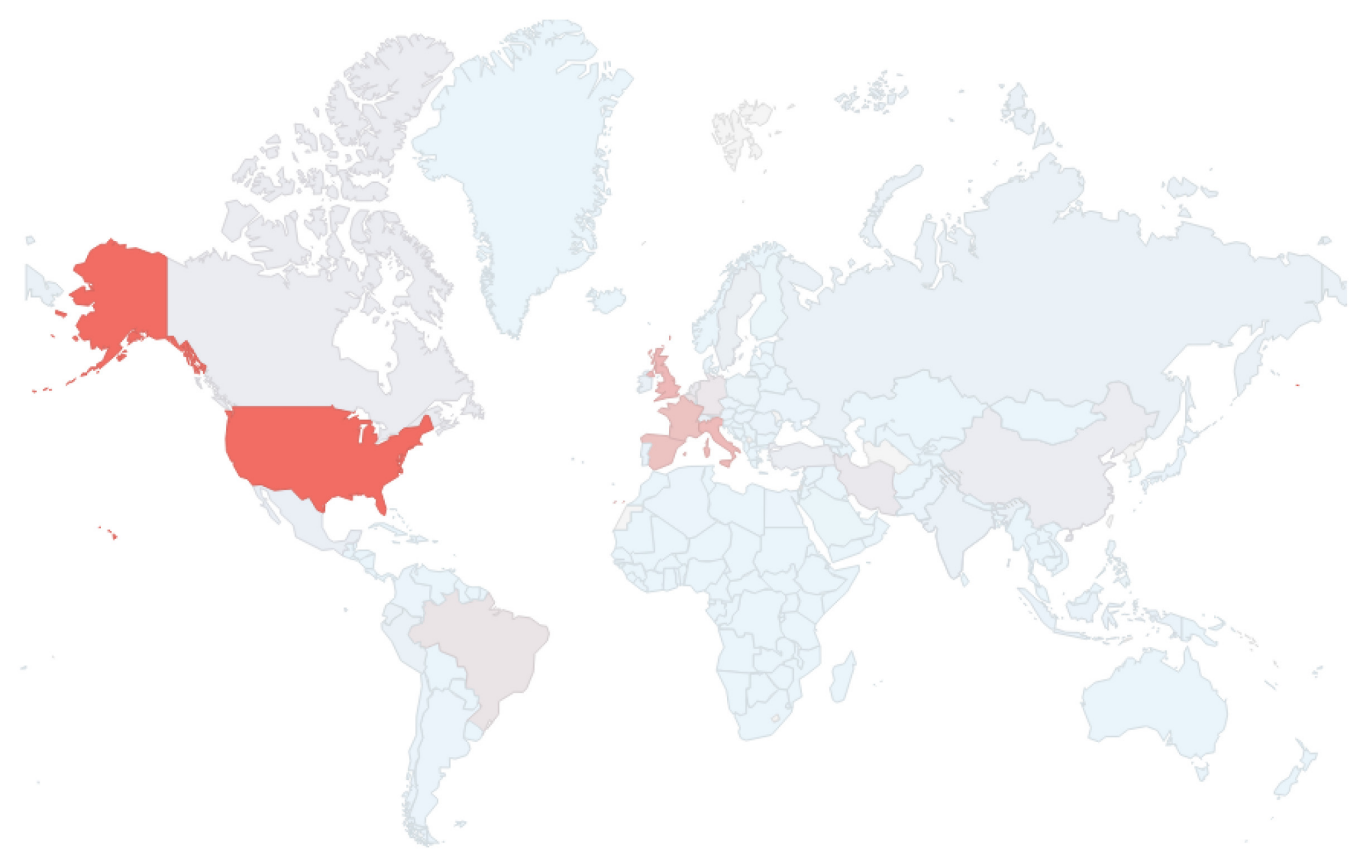
Geographic chart from *COVID-19 Dashboard* showing the number of deaths by country using colour coding. Red = high number of deaths; light blue = low number of deaths. Chart captured on May 29 ^th^ 2020.

As mentioned above, the number of cases, deaths, and mortality rates are deployed using geographic charts. Figure 2 presents a geographic chart with the number of deaths by country, where red implies a large number of deaths and light blue implies a low number of deaths. On the date that this chart was obtained from *COVID-19 Dashboard*, the United States had the largest number of deaths worldwide followed by Spain, France, Italy and the United Kingdom. However, just using the color coding, it is not clear if Spain, France, Italy or the United Kingdom has the second highest number of deaths. Similarly, for the lower number of deaths seen in Belgium, Brazil, Germany, Canada, Turkey, Netherlands, Iran, and China, it is not clear which has the lowest number of deaths using the color coding.

**Figure 3.**
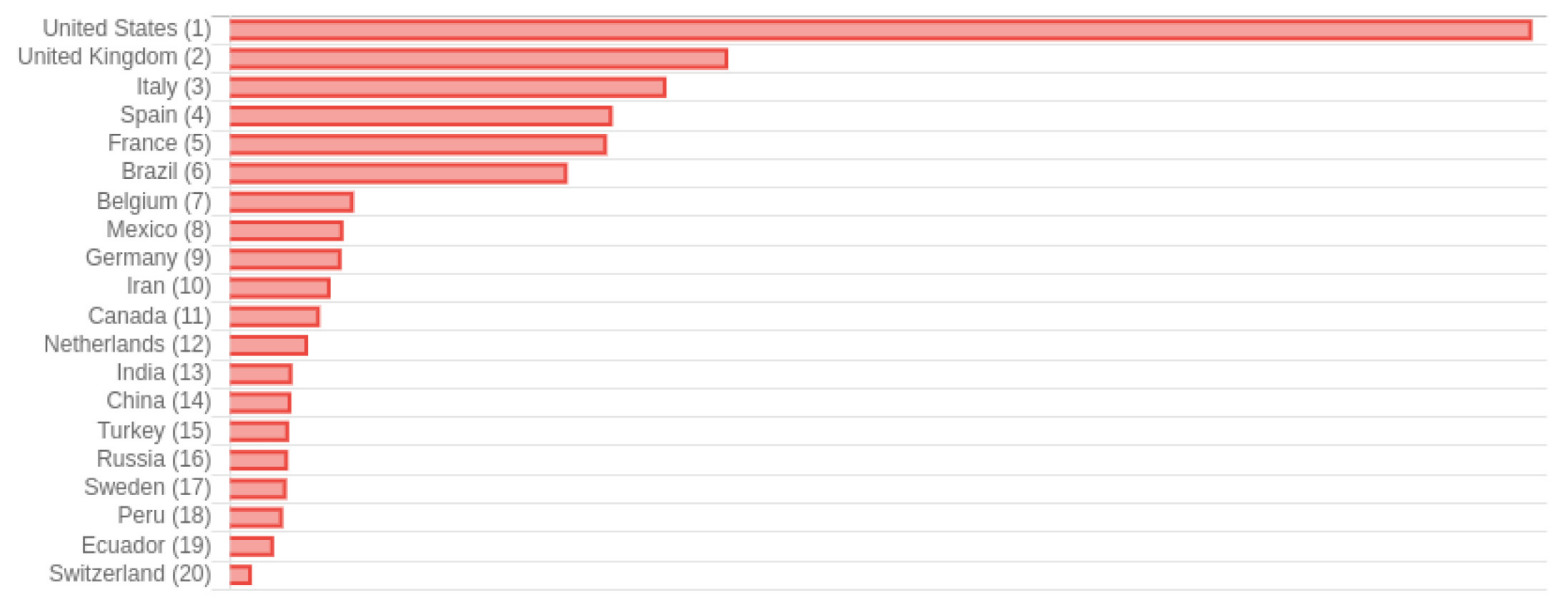
Fragment of a bar chart from *COVID-19 Dashboard* showing the number of deaths per country sorted from highest to lowest. Chart captured on May 29 ^th^ 2020.

Consequently, it is necessary to sort the countries based on the actual number of deaths in addition to the color coding. Figure 3 presents a fragment of a bar chart with the number of deaths per country sorted by number of deaths. This chart shows that the United States has more than the double of deaths of Italy and the United Kingdom. In addition, Spain and France have almost the same number, but a little less than Italy and United Kingdom, followed by Brazil, while Belgium, Mexico, Germany, Iran, and Canada have similar number of deaths but much less than Spain and France. Finally, Netherlands, India, China, Turkey, Russia, and Sweden have less but a still important number of cases.

**Figure 4.**
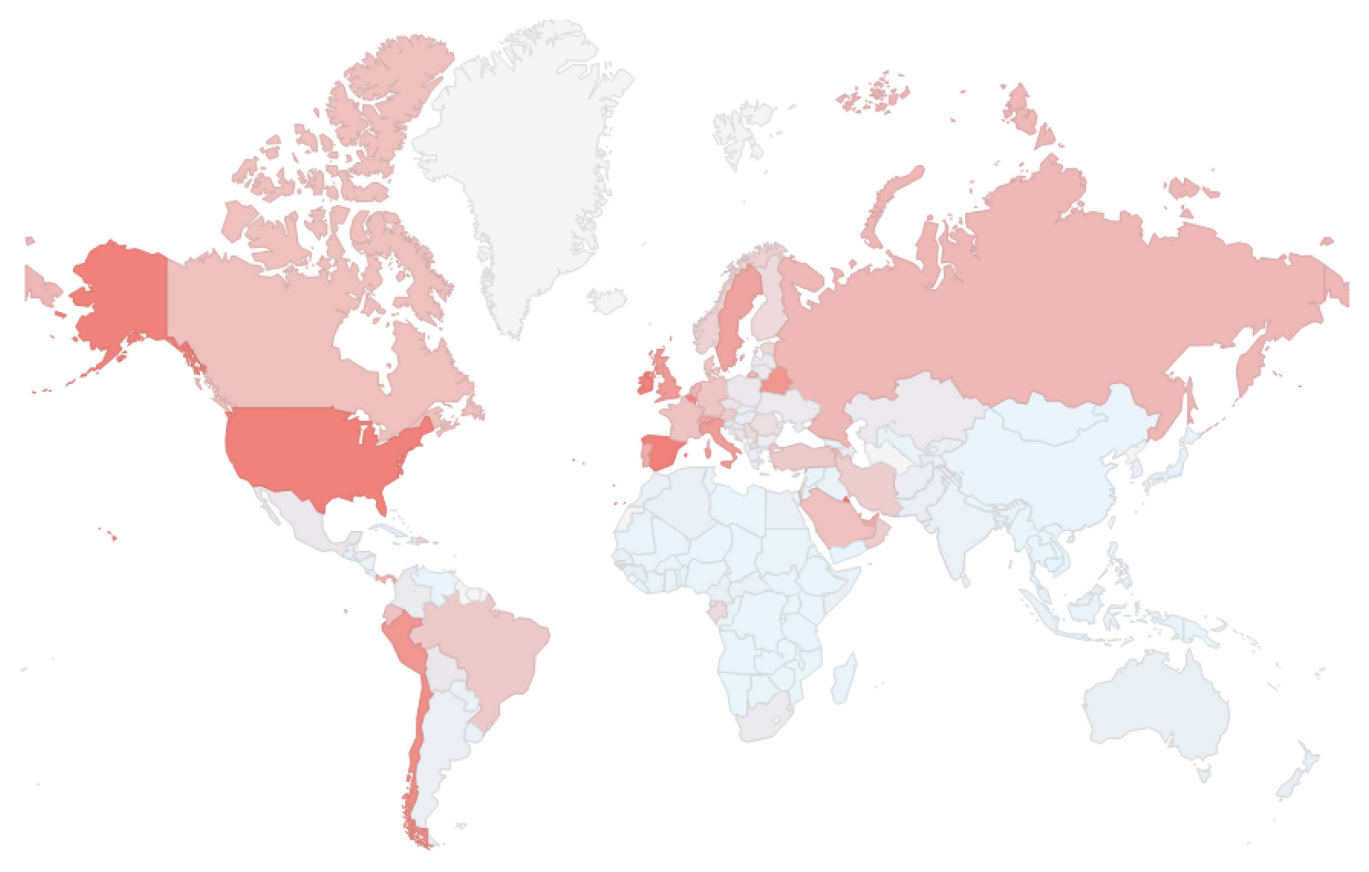
Geographic chart from *COVID-19 Dashboard* showing the number of cases by country per million people using color coding. Red = high number of deaths; light blue = low number of deaths. Chart captured on May 29 ^th^ 2020.

It is also important to assess information about COVID-19 based on the population by country. Figure 4 presents a geographic chart with the number of cases by countries per million people. Although United States has the largest number of cases, this chart presents that countries such as Spain, Ireland, and Belgium have more cases per million than United States, while Italy, Switzerland, United Kingdom, Portugal, and Sweden have also a large number of cases per million.

**Figure 5.**
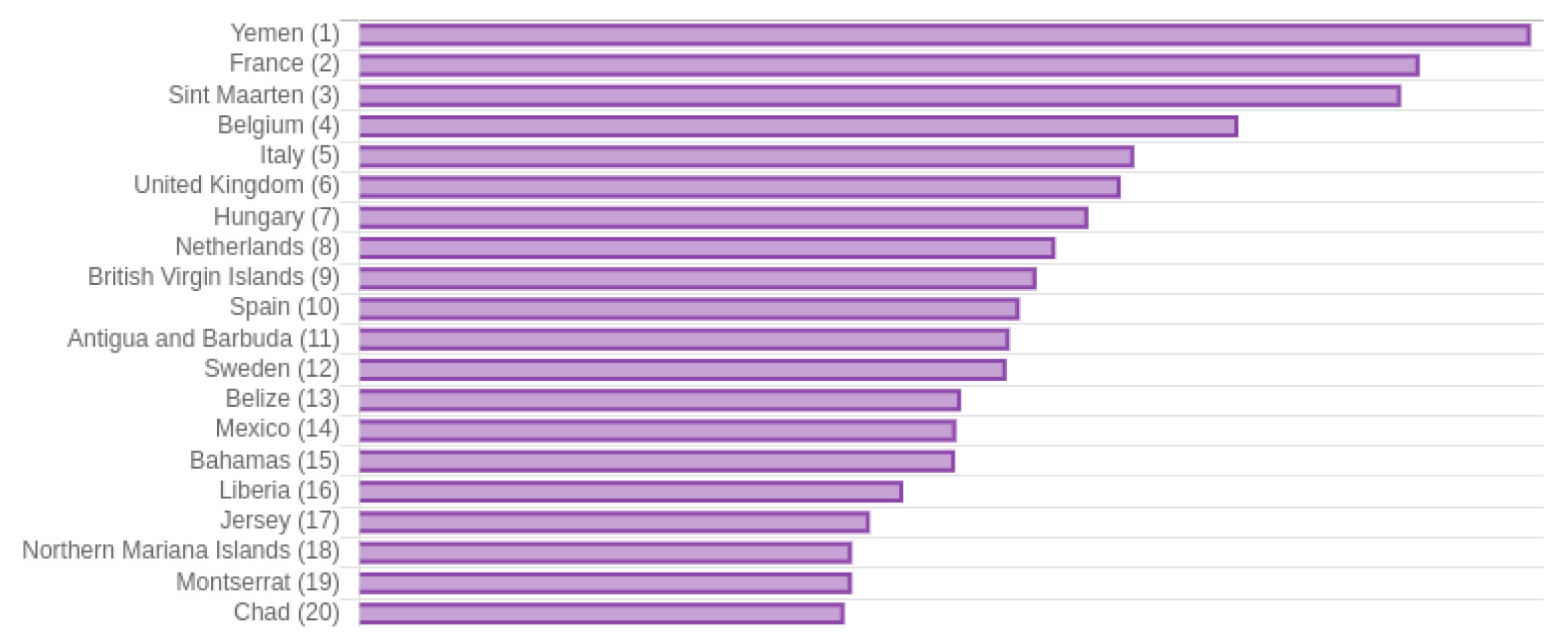
Fragment of bar chart from *COVID-19 Dashboard* showing mortality rates per country sorted highest to lowest. Chart captured on May 29 ^th^ 2020.

Additionally, the *COVID-19 Dashboard* shows the mortality rate per country, as shown in Figure 5. Although the United States has the largest number of deaths, it also has a very large number of cases. However, countries such as Italy, United Kingdom, Spain, and France have a higher mortality rate than the United States. Surprisingly, the country with the highest mortality rate by May 29 ^th^ is Yemen (21.63%), showing the very serious public health issue for this country.

Nevertheless, it is important to take into account that this country has reported little information. Countries such as France, Belgium, Italy, United Kingdom, and Spain are in the top 10 of this list, which implies that they have a high mortality risk, perhaps due to the high number of active cases.

**Figure 6.**
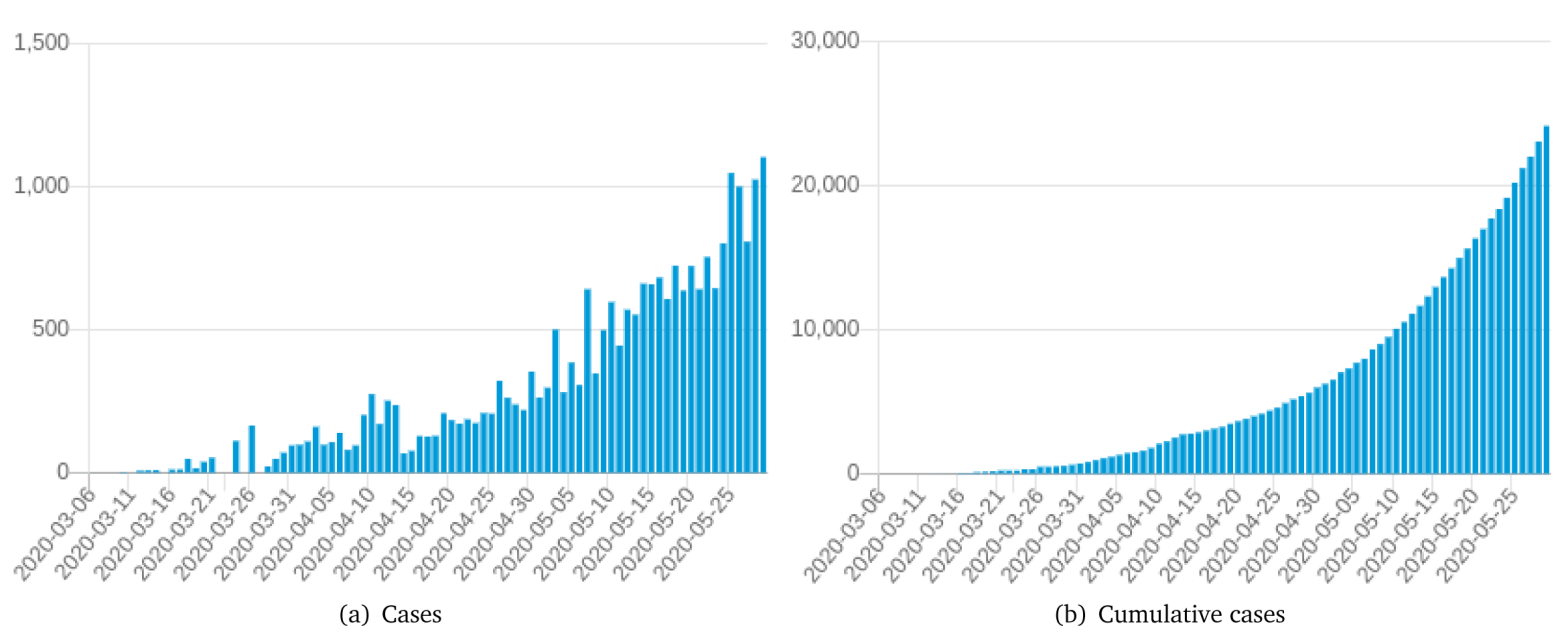
Bar chart from *COVID-19 Dashboard* showing cases per date in Colombia. Chart captured on May 29 ^th^ 2020.

Further information to be analyzed is cases and deaths per date. Figure 6 presents two charts. Figure 6(a) shows the number of confirmed cases per day in Colombia, while (b) shows cumulative cases per day in Colombia. Figure 6(a) shows that from April 1 2020, the number of cases was stable with peaks in the last few days, while Figure 6(b) shows stable behavior with a small increment of cumulative cases from April. It implies that COVID-19 keeps impacting health in Colombia and cases will increase until its behavior changes.

**Figure 7.**
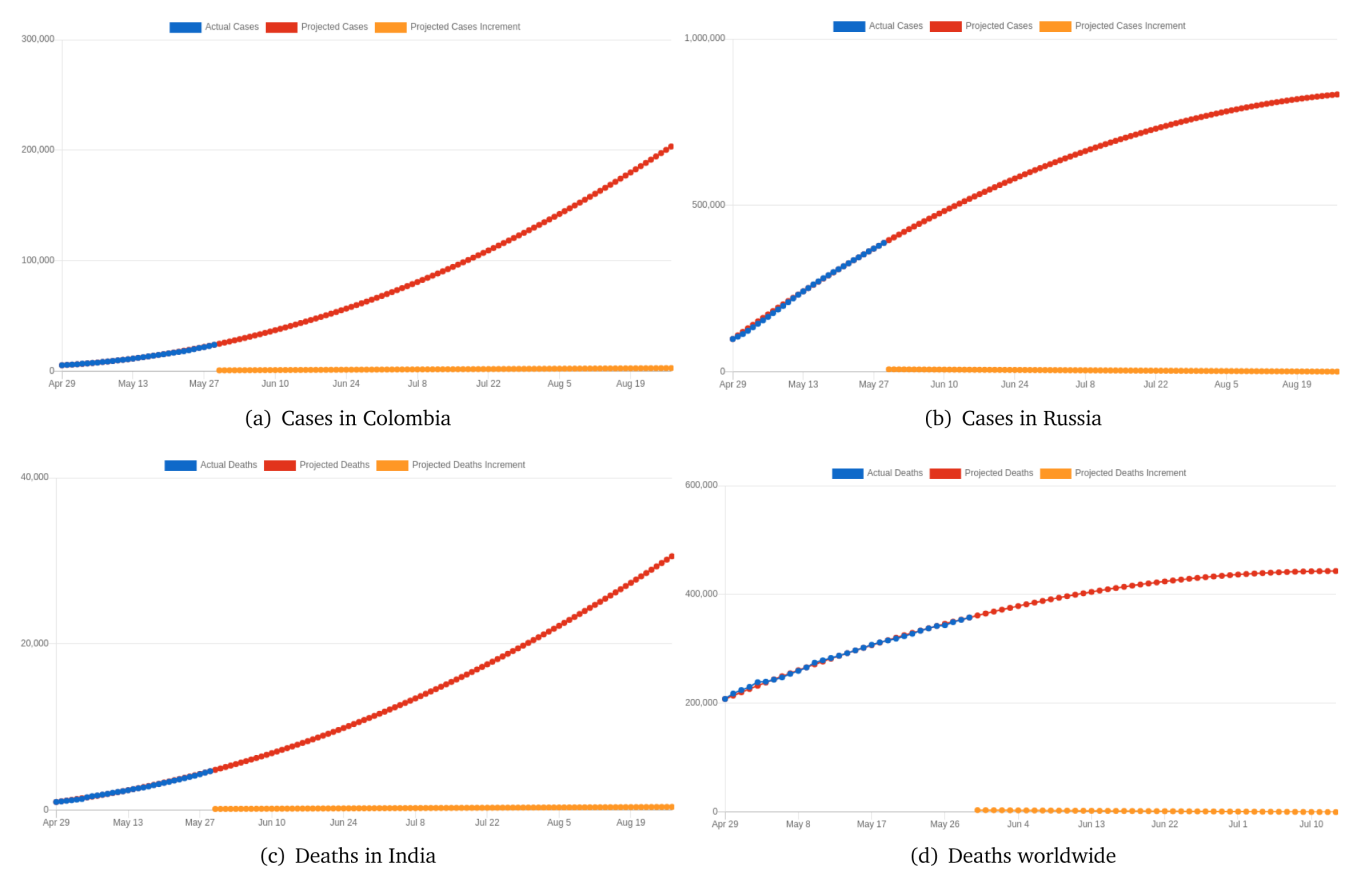
Cases and death projections from *COVID-19 Dashboard*. Chart captured on May 29 ^th^ 2020.

Based these previous results, it is really important to know the trend of cases and deaths worldwide and by country. Thus, *COVID-19 Dashboard* includes a very important contribution, i.e. the mathematical model already described, which allows projection for future cases and deaths worldwide and by country. Figure 7 presents four charts with cases and death projections.

- Figure 7(a) presents projected cases in Colombia. Blue corresponds to the actual cases reported by the WHO, while red corresponds to the projected cases and yellow the increment of cases per day. This projection performed on May 29 ^th^ 2020 produced the quadratic equation:
  $$f\left(x\right)=11.46x^{2}+273.10x+5,597.00\;\left(11\right)$$
  This projection presents a exponential growth, which implies that COVID-19 in Colombia will continue to impact public health.
- Figure 7(b) presents projected cases in Russia. This projection performed on May 29 ^th^ 2020 produced the quadratic equation:
  $$f\left(x\right)=-38.82x^{2}+10,772.00x+99,399.00\;\left(12\right)$$
  This projection presents a logarithmic growth, which implies that deaths for COVID-19 in Russia are decreasing considerably and might stop in 61 days.
- Figure 7(c) presents projected deaths in India. This projection performed on May 29 ^th^ 2020 produced the quadratic equation:
  $$f\left(x\right)=1.37x^{2}+82.30x+1,007.00\;\left(13\right)$$
  This projection presents a exponential growth, which implies that deaths for COVID-19 in India are still increasing.
- Figure 7(d) presents projected deaths worldwide. This projection performed on May 29 ^th^ 2020 produced the quadratic equation:
  $$f\left(x\right)=-41.22x^{2}+6,224.23x+208,111.00\;\left(14\right)$$

This projection presents a logarithmic growth, which implies that deaths for COVID-19 worldwide might start decreasing in the first week of July.

Quadratic equations are presented with coefficients with two decimals; however, all calculations are done with the real calculated values for their coefficients.

## Discussion

COVID-19 has required analysis of data regarding cases and deaths reported worldwide. Some projects developed by important organizations such as *COVID-19 Dashboard by the Center for Systems Science and Engineering (CSSE) at Johns Hopkins University (JHU)* ( https://coronavirus.jhu.edu/map.html) and *Coronavirus Disease (COVID-19) Dashboard by the World Health Organization* ( https://covid19.who.int/) have provided very interesting visualizations for reporting the current state of the COVID-19. Nevertheless, these projects lack important information and analysis. With this in mind, the *COVID-19 Dashboard* presented here has been designed and developed in order to offer additional valuable information. This project uses the data reported by the WHO; therefore, it is able to deploy the same information as the WHO COVID-Dashboard. However, *COVID-19 Dashboard* is able to deploy more information such as cases and deaths per million people worldwide and mortality rate worldwide, which is also very important to assess the impact of the disease from different perspectives.

Moreover, *COVID-19 Dashboard* has an additional important contribution not presented in other projects. This is the mathematical model that projects future cases and deaths worldwide and by country. This mathematical model is based on linear algebra and calculates a quadratic equation based on the information reported in the last 31 days. Thus, the mathematical model is able to adjust the quadratic equation once new information is reported. With this model, *COVID-19 Dashboard* is able to estimate the number of new cases and deaths up to 90 days.

## Conclusions

*COVID-19 Dashboard* allows understanding COVID-19 behavior and evolution from different perspectives. Based on those perspectives, it is possible to assess not only the actual number of cases and deaths worldwide and by country, but also the most impacted countries by observing the number of cases and deaths per million people and mortality rate. *COVID-19 Dashboard* offers a projection of future cases and deaths, which is an important tool to estimate the disease’s evolution. Based on these projections, health organizations around the world might take action in order to minimize the impact, as well as estimate the date when the COVID-19 might stop impacting various countries.

## Data availability

### Underlying data

Data used for this project has been taken from the following public sources available in JSON and Comma-separated values (csv) format respectively:

- COVID-19 map data by the WHO https://covid19.who.int/.
- Population by country 2020 data by the WPR https://www.worldpopulationreview.com/countries/.

## Software availability

- Software available from: https://covid19.itiud.org/.
- Source code available from: https://gitlab.com/florezfernandez/COVID-19Dashboard
- Archived source code at time of publication: https://doi.org/10.5281/zenodo.3825939 ^14^
- License: GNU General Public License (GPL)
