# Supplementary material for: Online dashboard and data analysis approach for assessing COVID-19 case and death data
Source: F1000Res. 2020 Jun 8;9:570. [Version 1] doi: 10.12688/f1000research.24164.1 (PMC7450472; doi:10.12688/f1000research.24164.1)
Supplement: Peer review history

# Peer review: Reviewer response for version 1

**DOI:** 10.5256/f1000research.26654.r64488

**Published:** 2020-08-26

**Authors:** Hüseyin Bicen

**Affiliations:** 1 Faculty of Open and Distance Education, Near East University, Nicosia, Cyprus

The paper is significant. The paper result is have an important data analysis approach for assessing COVID-19 case and death data. The Authors analyze the current state of COVID-19, have created an online information system called *COVID-19 Dashboard*, which is accessible from: https://covid19.itiud.org/. The paper result is relevant to the researchers. For that reason, this paper is necessary to the scientific world.

Are the conclusions about the tool and its performance adequately supported by the findings presented in the article?

Yes

Is the rationale for developing the new software tool clearly explained?

Yes

Is the description of the software tool technically sound?

Yes

Are sufficient details of the code, methods and analysis (if applicable) provided to allow replication of the software development and its use by others?

Yes

Is sufficient information provided to allow interpretation of the expected output datasets and any results generated using the tool?

Yes

Reviewer Expertise:

Educational Technology

I confirm that I have read this submission and believe that I have an appropriate level of expertise to confirm that it is of an acceptable scientific standard.

# Peer review: Reviewer response for version 1

**DOI:** 10.5256/f1000research.26654.r64489

**Published:** 2020-06-26

**Authors:** Olmer Garcia-Bedoya

**Affiliations:** 1 Jorge Tadeo Lozano University, Bogota, Colombia

The authors present a Dashboard's development to analyze the COVID-19 contagious and death base in open data available. 

 The paper is well-written and presents interesting results clearly and concisely. I have enjoyed reading this paper.

 I have the following comments for the authors that should be addressed before indexing:

- The model presented to predict the time series's tendencies is easy to understand and has a fast implementation. However, I recommend reviewing time series analysis like ARIMA, recurrent neural network, or systems dynamics, which let to include more data in the estimation of the tendencies of the time series.
- It will be interesting to review initiatives like presented in Kaggle wherein collaborative works, have presented alternatives since the data science field could help to combat the COVID-19 disease.

Are the conclusions about the tool and its performance adequately supported by the findings presented in the article?

Partly

Is the rationale for developing the new software tool clearly explained?

Yes

Is the description of the software tool technically sound?

Yes

Are sufficient details of the code, methods and analysis (if applicable) provided to allow replication of the software development and its use by others?

Partly

Is sufficient information provided to allow interpretation of the expected output datasets and any results generated using the tool?

Yes

Reviewer Expertise:

Robotics and data science Professor

I confirm that I have read this submission and believe that I have an appropriate level of expertise to confirm that it is of an acceptable scientific standard, however I have significant reservations, as outlined above.

# Peer review: Reviewer response for version 1

**DOI:** 10.5256/f1000research.26654.r64490

**Published:** 2020-06-24

**Authors:** Maria Florencia Pollo-Cattaneo

**Affiliations:** 1 Grupo GEMIS, Facultad Regional Buenos Aires Universidad Tecnologica Nacional, Buenos Aires, Argentina

This paper presents a proposal for a COVID-19 Dashboard to manage significant and relevant information in relation to the pandemic that is currently attacking the whole world.

 From a methodological point of view, the solution is developed with solidity and technologies adapted to the technological problem posed.

 The resulting product is tested with valid data even though it is not developed and analyzed in all countries in detail due to the extension of the work.

 It would be interesting if they could update and validate if the data obtained by the board match the real data and see if they could take into account second outbreaks of the disease.

 The solution is validated correctly.

Are the conclusions about the tool and its performance adequately supported by the findings presented in the article?

Yes

Is the rationale for developing the new software tool clearly explained?

Yes

Is the description of the software tool technically sound?

Yes

Are sufficient details of the code, methods and analysis (if applicable) provided to allow replication of the software development and its use by others?

Partly

Is sufficient information provided to allow interpretation of the expected output datasets and any results generated using the tool?

Yes

Reviewer Expertise:

artificial intelligence - informatic -  information systems

I confirm that I have read this submission and believe that I have an appropriate level of expertise to confirm that it is of an acceptable scientific standard.
